# Variational and Deep Learning Segmentation of Very-Low-Contrast X-ray Computed Tomography Images of Carbon/Epoxy Woven Composites

**DOI:** 10.3390/ma13040936

**Published:** 2020-02-20

**Authors:** Yuriy Sinchuk, Pierre Kibleur, Jan Aelterman, Matthieu N. Boone, Wim Van Paepegem

**Affiliations:** 1 Department of Materials Science and Engineering, Faculty of Engineering and Architecture, Ghent University, Technologiepark Zwijnaarde 46, 9052 Zwijnaarde, Belgium; wim.vanpaepegem@ugent.be; 2 Department of Environment, Faculty of Bioscience Engineering, Ghent University, Coupure Links 653, 9000 Gent, Belgium; pierre.kibleur@ugent.be; 3 Center for X-ray Tomography (UGCT), Ghent University, Proeftuinstraat 86, 9000 Gent, Belgium; jan.aelterman@UGent.be (J.A.); matthieu.boone@UGent.be (M.N.B.); 4 Department of Telecommunications and Information Processing–Image Processing and Interpretation, Faculty of Engineering and Architecture, Ghent University—IMEC, Sint-Pietersnieuwstraat 41, 9000 Gent, Belgium; 5 Department of Physics and Astronomy, Faculty of Sciences, Ghent University, Proeftuinstraat 86, 9000 Gent, Belgium

**Keywords:** fabrics/textiles, carbon-fiber reinforced polymer, multi-scale modelling, image segmentation, microcomputed tomography

## Abstract

The purpose of this work is to find an effective image segmentation method for lab-based micro-tomography (µ-CT) data of carbon fiber reinforced polymers (CFRP) with insufficient contrast-to-noise ratio. The segmentation is the first step in creating a realistic geometry (based on µ-CT) for finite element modelling of textile composites on meso-scale. Noise in X-ray imaging data of carbon/polymer composites forms a challenge for this segmentation due to the very low X-ray contrast between fiber and polymer and unclear fiber gradients. To the best of our knowledge, segmentation of µ-CT images of carbon/polymer textile composites with low resolution data (voxel size close to the fiber diameter) remains poorly documented. In this paper, we propose and evaluate different approaches for solving the segmentation problem: variational on the one hand and deep-learning-based on the other. In the author’s view, both strategies present a novel and reliable ground for the segmentation of µ-CT data of CFRP woven composites. The predictions of both approaches were evaluated against a manual segmentation of the volume, constituting our “ground truth”, which provides quantitative data on the segmentation accuracy. The highest segmentation accuracy (about 4.7% in terms of voxel-wise Dice similarity) was achieved using the deep learning approach with U-Net neural network.

## 1. Introduction

The development of realistic models of woven composite material for meso-scale simulations has grown significantly over the last decades. Existing approaches for generation of the material finite element model can be grouped roughly in three categories: (1) creation of idealized geometry based on statistical data (e.g., analytical representation of a tow surface); (2) extraction of geometry from the result of a numerical simulation (e.g., simulation of fabric compaction); (3) development of a model based on real micro-tomography (µ-CT) images.

Formation of the idealized geometry of a tow is the most evident method to get a model. Several software solutions are developed for this purpose; the most known are TexGen [1] and WiseTex [2]. Unfortunately, the creation of an idealized geometry, which is close to the real shape, is often difficult. The resulting volume of idealized tows is often less than the real one. Artificially increasing the fibers volume fraction within a tow is used to preserve correct overall material properties, which in turn can have an undesirable effect on the simulation result [3].

The second approach generates the textile geometry from a simulation aimed to get a real shape model, such as the mechanical modelling of the material compaction and forming. In the work [4], the tows in textiles are represented as chains of 1D rods, which are put in contact to represent the textile geometry. This approach was further developed in study [5], where tows are modelled as multi-chain bundles. Similarly, the work [6] demonstrated a simulation-based geometry obtained by using the digital element method (where virtual fibers are chains of truss elements). Such approaches have been validated by comparison to CT data. For example, in the work [7], the resulting geometry is sufficiently close to the reference µ-CT data. However, the difficulty of such methods could be the adjustment of the model parameters needed to get the desired shape. In general, this approach could be considered as an improvement of the existing idealized geometry, since the simulation always has to start from the initial (idealized or dry) state of tows. For example, in the work [8] the dry fabric compaction is simulated in order to generate textile geometries starting from the output of TexGen software.

The third possibility is modelling textile geometry based on µ-CT data [9,10]. The procedure includes two steps: µ-CT image processing and image-based meshing. Presently, the input µ-CT data is often acquired at the limits of the scanning device capabilities, to reach the highest possible image quality. Yet, typical complexity of processing such images involves the relatively low resolution (which is a trade-off with the sample size), noise, and the low contrast between different material constituents (i.e., carbon and resin). An alternative way to automatized segmentation, manual segmentation, is prohibitively time-consuming due to the size of these datasets [3,11].

State-of-the-art image processing applied to these datasets is fiber orientation analysis with the subsequent application of a segmentation method or machine learning without pre-processing. In the work [12], the structure tensor approach is used for the fiber orientation analysis based on the µ-CT images. This method uses a clever averaging of the image gradients to robustly determine orientation and anisotropy level. However, the structure tensor analysis for a noisy µ-CT or low contrast image is less adequate, mainly because of the small difference in detectable anisotropy between tow and matrix regions. Applied to carbon fiber reinforced polymers (CFRP) datasets, the fiber orientation analysis result leads to a very noisy bicolor image. The segmentation of such images is a fundamental problem of image processing and computer vision. So far, many advanced segmentation techniques have been developed: watershed transformation [13], partial differential equation-based methods [14], model-based segmentation [15], graph partitioning [16,17], trainable segmentation [18], and variational methods [19]. The goal of the variational approach is to find a segmentation that is optimal with respect to a specific energy functional. The work [19] demonstrates the efficiency of such techniques, applied to the segmentation of 2D colored images, as well as high performance video smoothing using graphics processing unit (GPU) parallelization.

In the present work, we followed and developed the µ-CT image-based modeling approach. The goal is to find an effective image segmentation method for µ-CT data of CFRP. This material is comprised of a plain woven tows architecture at the meso-scale with low contrast-to-noise ratio. The segmentation of this dataset is challenging because of the following reasons: firstly, the typical µ-CT resolution is too low with respect to the fiber diameter to identify these fibers; secondly, data are quite noisy, partly due to phase contrast effects at the fibers (resulting in a speckle on the projection data) and, finally, µ-CT contrast between the material constituents is too low (similar mean value of gray level for carbon and resin regions). We, therefore, propose to solve this problem with two strategies: the first using a variational method and the second using deep learning. As far as the authors are aware, it is entirely novel to apply these methods to µ-CT data of CFRP woven composites.

The variational method requires the extraction of the fiber orientation, yielded by the application of morphological operations on the raw µ-CT data [20]. The fiber orientation analysis resulted in two images of orthogonally-arranged tows: the warp and the fill directions. Afterwards, these orientation images were subsampled and segmented using the method [19], which is based on the minimization of the Mumford–Shah functional [21].

The alternative deep learning approach, based on a convolutional neural network with U-Net architecture [22], was used to provide the segmentation from an input. We have evaluated both a technique that uses the gradient images as input as well as the raw images.

Overall, the solution of the segmentation problem proposed in this paper, based on the above-mentioned recent achievements in the computer vision field, could significantly extend the range of images that can be used for automatic generation of the realistic textile geometry.

The paper has the following structure: in Section 2, general characteristics of the investigated material and its µ-CT data are presented. Section 3 describes the method to manually create a ground truth segmentation. The method for the fiber orientation analysis is presented in Section 4. The segmentation procedures are described in Section 5 and Section 6 (starting from the raw and the orientation images). Section 7 describes the validation of the automatic segmentation results. The discussion is drawn in Section 8. Section 9 summarizes the outcomes of this study.

## 2. Material Characterization

**Figure 1:**
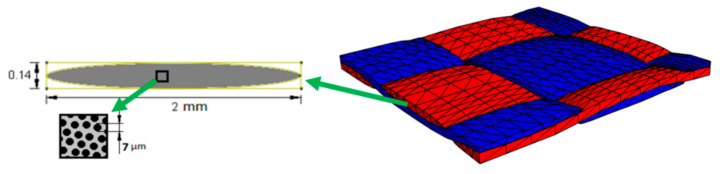
Tows geometry at the micro- and meso-scales.

The material under investigation is a plain-weave CFRP laminate (TR3110 360GMP, Mitsubishi Rayon Co., Tokyo, Japan) with layup [#(0/90)]_8_. It has 8 plies of regular woven fabric with a ply thickness of 0.23 mm (the total thickness ~1.8 mm) and count ends (warp/fill tows) per inch length of 12.5. Each tow contains 3000 continuous fibers with a diameter of about 7 µm. The schematic illustration of the microstructure geometry at the micro- and meso-scales is presented in Figure 1. The single ply (Figure 1) shows the material geometry at meso-scale created by manual processing of the real image (see Section 3 for more details).

The material is periodic in-plane with equal periodic lengths in the warp and fill directions: $2\;\times \frac{25.4}{12.5}=4.064\;\mathrm{mm}$ . From the stack thickness and periodic lengths, we retrieve the minimum image size that contains the complete unit cell (UC) geometry: about 2 × 5 × 5 mm³, which imposes a limit on the resolution of the µ-CT image. It is usually good to have an image that is slightly larger than a single UC, to avoid edge effects on the image processing routine.

**Figure 2:**
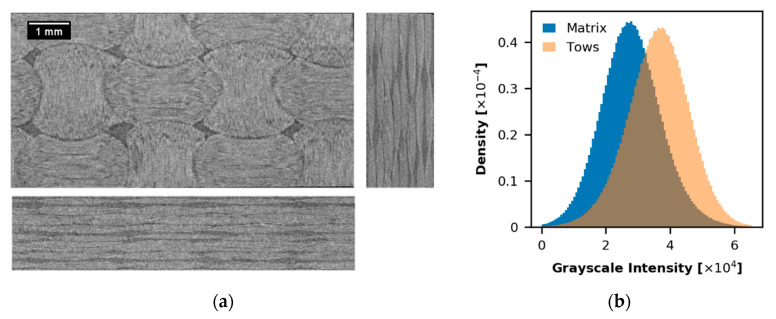
(**a**) Top, front, and side (reconstructed) slices of the input µ-CT image (size in voxels: 368 × 972 × 1723, physical size: 1.8 × 4.9 × 8.6 mm^3^, voxel size ~5 µm); (**b**) Intensity histograms.

The material was scanned at the custom-designed µ-CT system HECTOR of the Ghent University Centre for X-ray Tomography (UGCT, Gent, Belgium) [23]. Three orthogonal µ-CT slices of the material are presented in Figure 2. The µCT dataset is a 16-bit unsigned integer array with dimensions (368, 972, 1723) pixels that corresponds to the physical size of 1.8 × 4.9 × 8.6 mm³. The scan holds an array of 1 × 2 UC. The gray value in each voxel corresponds with a local linear attenuation coefficient through a slope and offset applied to the unsigned integer value. Each voxel represents a physical volume of ~5 × 5 × 5 µm^3^ (note that the voxel size is close to the fiber diameter). Figure 2a shows that the intensity of matrix and tow voxels is similar and the local fiber orientation (texture anisotropy) is not clearly defined. Here, the fiber orientation is extracted only in-plane (fill and warp directions). Visually, it can be identified as the direction of darker lines in tows’ area which comes from rich resin regions. Moreover, the tows’ region has a locally smoother grey transition in the fill or warp fiber direction.

For noise quantification, the mean ( μ ) and standard deviation ( σ ) of matrix and tows regions were calculated using the manual segmentation of a single UC (presented in Section 3). The following values were obtained: $\mu_{M}=27,854;\;\sigma_{M}=9573;\;\mu_{T}=36,155;\;\sigma_{T}=9682$ . The matrix mean lies within 1-sigma of the tows mean and vice versa. The signal-to-noise ratios can be evaluated as $\mu_{M}/\sigma_{M}=\;2.9$ and $\mu_{T}/\sigma_{T}=\;3.7$ .

## 3. Manual Segmentation

**Figure 3:**
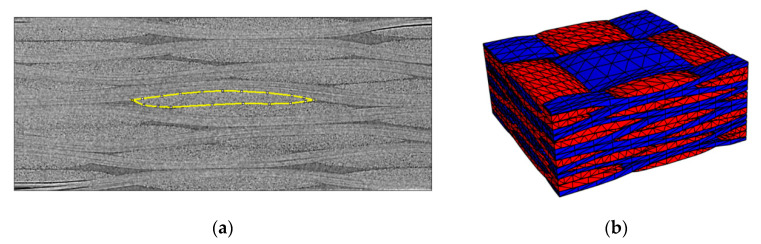
The manual segmentation steps: (**a**) Drawing a tow cross-section contour; (**b**) Tow surfaces from the cross-section contours.

The raw µ-CT image was segmented manually, for quality evaluation of the tow labeling results. This reference “ground truth” segmentation was created by tow cross-section selection, using the ImageJ software (1.52, University of Wisconsin, WI, USA) [24]. Firstly, the raw image (and in time, so was the result of segmentation) was cropped to 357 × 862 × 861 voxels, from the initial 368 × 972 × 1723. At this size, the complete UC geometry is still captured, but it allows us to halve the work of the manual segmentation. Moreover, cropping eliminates the boundary effects in automatic segmentation results, making the comparison more representative of the bulk of the material. Next, the cropped image was scaled down for each of the tow directions using averaging, which results in two datasets with sizes 357 × 111 × 861 and 357 × 862 × 111. This subsampling reduces noise, aiding the visual identification of tow edges. The importance of this can be seen when comparing Figure 3a with the raw image in Figure 2a. Then for each tow, 12 cross-section contours were created as shown in Figure 3a. Overall, the ground truth consists of 24 tows in one direction and 22 in the other, for a total of 552 contours. The resulting contours were used to generate tow surfaces in the original size (Figure 3b). The algorithm developed by the authors, as well as freely available Python packages (including the VTK 8.2.0 library wrapper [25]), were used for the surface processing operations. All tow surfaces were rasterized using the ITK 5.0.1 library [26] and joined to a single labeled image. The voxels of tow intersection regions were assigned to the closest tow color using the chamfer distance transform. Afterwards, all isolated matrix regions that were small (volume <10^5^ voxels) were removed by the same procedure used in removing tow intersections. The threshold was selected quite large to avoid having too many finite elements in the mesh.

The procedure of the manual segmentation is summarized in Algorithm 1. Note, the tows must be aligned parallel to the image axis. For joining tows’ images, it uses an auxiliary procedure that joins binary images (see code in Appendix A). A slice image of the manual segmentation is shown in results of Section 7.

**Algorithm 1: Manual Segmentation.**

(1) Do steps 2–5 for warp and fill directions (independently).
(2) Downsample the image in the direction of the corresponding tow.
(3) Create cross-section contours manually. The “Polygon selections” function of ImageJ is used here.
(4) Generate tow surfaces from the input contours (with the original size in the direction of the tow). Here the surfaces were created by inhouse code, which connects the contours’ nodes by lines (each contour has the same count of nodes).
(5) Rasterize tow surfaces (for each tow it gives us a 3D image with the original image size).
(6) Join individual tows’ images using function *join_images* with threshold parameters 10^5^ (code in Appendix A).

## 4. Fiber Orientation Analysis

The fiber orientation was analyzed based on the image texture gradients. Two main orientations (warp and fill direction) were recognized. These orientations coincide with the gradient direction (in-plane of the material sample). Potential out-of-plane fiber orientation change was ignored at this stage of the processing because it is negligibly small.

**Figure 4:**
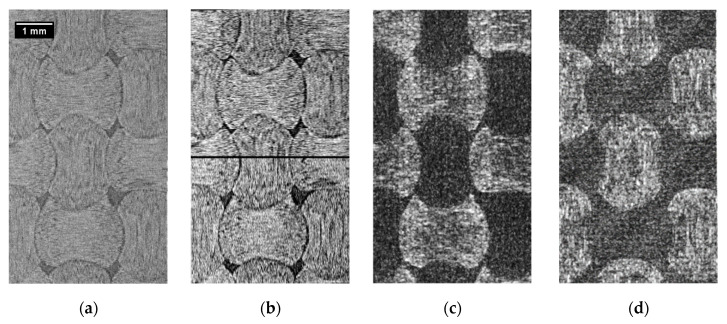
(**a**) Raw image; (**b**) Effect of the uniform filter for horizontal (top) and vertical (bottom) direction of the kernel; (**c**) Morphological gradient for vertical and (**d**) horizontal orientation of the line structure element.

Smoothing along the fiber direction was performed before calculating the gradient in the transverse to the fibers’ direction. Here, a uniform 1D filter was used where all values within the line of the filter support have the same weight. The smoothing step improves the contrast of the gradient images (denoising texture in the fiber direction). The filter effect is shown in Figure 4b, where the results of the smoothing for both directions are combined in a single illustration. In this work, the morphological gradient was used for the orientation analysis. The method demonstrates a better result than classical gradient-based operators such as Sobel. In Figure 4c,d the resulting morphological gradient calculations are presented. These were calculated using an 11-voxel line structure element (which corresponds to the filter kernel used for the uniform filtering). The kernel size for both filters (11 voxels or ~55 µm) was chosen based on visual evaluation of the final images. For this purpose, the fiber orientation analysis was performed for different kernel sizes in the range from 3 to 21 voxels. Algorithm 2 describes shortly the steps of the fiber orientation analysis. It takes a raw image as an input and splits it into the fill and warp tow images. The images are named “gradient images”, as they correspond to directional differences of the local intensity.

**Algorithm 2: Fiber Orientation Analysis.**

(1) Do steps 2–3 for warp and fill directions (independently).
(2) Smooth the image by uniform (mean) 1D filter (function *uniform_filter1d* from the Python SciPy 1.3.0 library [27]). The kernel is oriented in the fiber direction (e.g., kernel size (0, 11, 0)).
(3) Calculate 1D morphological gradient with line structure element, oriented transverse to the orientation of the uniform filter kernel (e.g., kernel size (0, 0, 11)).

## 5. Image Segmentation by Variational Approach

The next processing step is the actual segmentation (isolation of a tow direction), performed on the results of the orientation analysis. The idea of the variational approach is to find a piecewise-smooth function approximation of the initial image by minimizing the Mumford–Shah functional [19,21]:

$$\underset{u,\;K}{\min}\left\{\int_{\Omega }^{}{\left|u-f\right|}^{2}dx+\alpha \int_{\Omega \backslash K}^{}{\left|\nabla u\right|}^{2}dx+\lambda \left|K\right|\right\},$$

where $\Omega \subset R^{d}$ is the image domain ( d=3 for the volumetric case); $f:\;\Omega \to R^{k}$ is the initial image function ( k=1 for gray-scale images); $u:\;\Omega \to R^{k}$ is the unknown approximation which is smooth everywhere in Ω but can have a jump at sub-regions’ edges K ; |·| is the Euclidean norm; α> 0 and λ> 0 are parameters which control the smoothness and length of edges. The functional contains three terms that simultaneously penalize: (1) The distance between the initial image and its approximation; (2) An approximation of the gradient norm (lack of smoothness) within sub-regions, and (3) the total length of the sub-regions’ edges.

**Figure 5:**
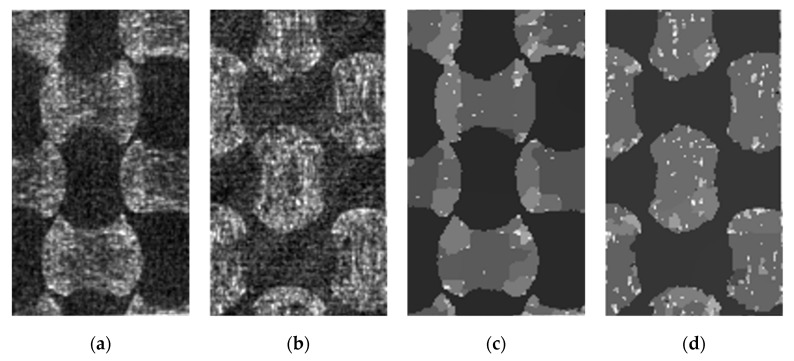
Downsampled results of the orientation analysis (**a**,**b**) and the corresponding results of Mumford–Shah functional minimization (**c**,**d**).

We remark that the minimization of the Mumford–Shah functional is a non-convex problem. In this work, the method proposed in the work [19] was used for solving it. It is based on an extension of a primal-dual approach from convex to non-convex optimization. In this study, the optimization algorithm was implemented for 3D gray-scale input (Figure 4c,d). Firstly, the in-plane image resolution was decreased by a factor of 10, while the out-of-plane resolution was preserved. Some such examples are shown in Figure 5a,b. This operation decreases the size of the input data by a factor of 100, from 368 × 972 × 1723 voxels to 368 × 97 × 172 voxels. We found that subsampling does not lead to major loss of information on the tows/matrix edges, as tow sections exhibit a large area and have smooth edges in-plane. Furthermore, data reduction enables the segmentation method to be completed on a standard laptop. Another advantage is that subsampling introduces a smoothing effect that increases the contrast between the sub-regions (tows/matrix).

The Mumford–Shah functional was minimized for the decreased resolution images and with a given set of parameters α and λ . The functional minimization algorithm is an iterative procedure [19]. It stops when the difference between two sequential segmentations is small enough: $\frac{1}{\left|\Omega \right|}\sum_{x\in \Omega }\left|u^{n+1}\left(x\right)-u^{n}\left(x\right)\right|<\varepsilon$ . The parameter ε controls the end of the iteration loop in case the segmentation is converged. In the work [19], it is shown that the iterative procedure always terminates if $\frac{1}{\left|\Omega \right|}\sum_{x\in \Omega }\left|u^{n+1}\left(x\right)-u^{n}\left(x\right)\right|\;\to 0$ , and the segmentation sequence $u^{n}$ is bounded for α< ∞ . The convergence of the sequence is not proven, but it is experimentally evaluated that the convergence rate is roughly $O\left(1/n^{2}\right)$ . The limiting case α→∞ imposes a zero gradient outside of sub-regions’ edges K . Therefore, the parameter α has to be large enough to ensure piecewise constant (not piecewise smooth) approximations of the input image. On the other hand, the increase of α could make the convergence slower. The parameter λ controls the count of small sub-regions in the resulting image (increasing λ decreases the count of sub-regions and vice versa). Theoretically, λ could take any positive value but λ ∈(0,1) was used in all of the successfully performed tests. In this work, the parameters $\alpha =10^{4}$ and λ=0.05 were chosen empirically, based on the visual evaluation. The results of the Mumford–Shah functional minimization are presented in Figure 5c,d.

**Figure 6:**
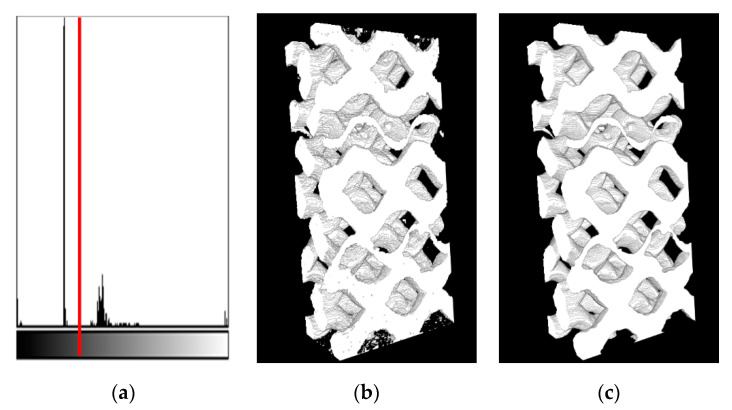
Postprocessing of Mumford–Shah functional minimization result (the illustrations correspond to Figure 5d): (**a**) The image histogram with red line threshold; (**b**) The thresholding result, and (**c**) cleaned result.

The threshold step was easily performed manually because the background voxels have very high contrast to the tows region (see Figure 6a). Here, the threshold value 65 was taken (the greyscale is from 0 to 255). For example, for the histogram in Figure 6a, the estimated upper limit of voxel count, which could be wrongly classified due to the manual threshold, is less than 0.88% of the total amount of voxels. Note, the splitting of such a histogram could also be performed automatically. The threshold step gives us an image with the small isolated and weakly connected regions (mostly close to the image boundary). These regions were handled during the cleaning step, which includes removing all isolated islands with count of voxels less than 1000 and the median filter application. The median filter had a kernel size of [3 × 5 × 5] voxels, which is small enough for preserving sharp matrix features. This size [3 × 3 × 3] is the smallest possible size of the 3D kernel and the size larger than 5 voxels can oversmooth the given tows structure of the downsampled image significantly. The effect of the cleaning step is shown in Figure 6b,c.

**Figure 7:**
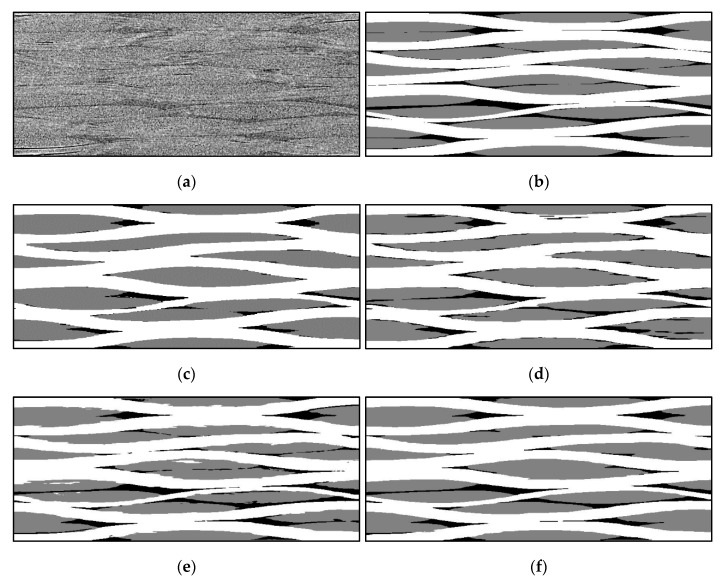
Demonstration of the segmentation results: (**a**) Raw image; (**b**) Ground truth; (**c**) Variational segmentation; (**d**) Deep learning for gradient images; Deep learning for raw image with 6 (**e**) and 14 (**f**) training slices.

These results were upsampled to the original image size. Then, the fill and warp images were combined into a single image. The resulting image usually has tows intersection regions. These regions and small isolated regions of the matrix were filled by colors of the closest tows’ voxels (function *join_images* in Appendix A). Corresponding slices of the segmentation result, raw image, and manual segmentation (see Section 7) are presented in Figure 7a–c. Algorithm 3 summarizes the described procedure of the variational segmentation. The processing by the variational approach takes about half an hour of computation time for the aforementioned volume using a standard workstation without GPU acceleration.

**Algorithm 3: Variational Segmentation.**

(1) Do steps 2–8 for warp and fill directions (independently).
(2) Analyze orientation (Algorithm 2).
(3) Downsample 10 times in-plane, while preserving the out-of-plane size (function *resize* from the Python Scikit-image 0.15.0 library [28]).
(4) Minimization of the Mumford–Shah functional using algorithm [19], with adopted parameters $\alpha =\;10^{4},\;\lambda =0.05$ and $\varepsilon =10^{-5}$ (see Section 5 for details on the choice of the parameters).
(5) Threshold (the value 65 was manually set using ImageJ software, as shown in Figure 6a).
(6) Remove small isolated regions (background and foreground) with voxel count less than 1000 (function *remove_small* in Appendix A).
(7) Median filter with kernel size [3 × 5 × 5] (function *median_filter* from Python SciPy library).
(8) Upsample back to the original image size (function *resize* from the Python Scikit-image library).
(9) Join warp and fill images using function *join_images* with threshold parameters 10^5^ (code in Appendix A).

## 6. Deep Learning Segmentation

A technique for segmentation of warp/fill tow directions and the matrix was developed with DragonFly (4.1, Object research systems (ORC) inc, Montreal, Canada) (free-of-charge for non-commercial use). We used part of the dataset that was manually segmented as training dataset, to train a convolutional neural network (U-net) on sporadically sampled slices. Out of a complete volume containing 357 slices (in-plane of material layers), we extracted 14 slices to constitute the training set. They were taken at 25 slices intervals to represent best the sample throughout, in spite of the low sampling rate.

In this work, only limited modification of the network structure was investigated. Different architectures were evaluated, but it soon became evident that the best results were obtained with the U-Net architecture. All U-Net networks that we trained had 32 layers and 22 × 10^6^ nodes. Fitting this approach to our research mostly involved changing the training parameters and learning set. Although we tried different learning parameters, we eventually reached the same conclusions that had been shared by Ronneberger et al. [22], i.e., that maximizing the patch size and minimizing the batch size to fit memory requirements yielded the best results. Indeed, the batch size parameter defines the number of patches that will be propagated through the network. It is closely related to the patch size parameter, in the sense that the patch size times the number of patches amounts to an array that needs to fit in GPU memory. Therefore, the parameters that we set are very specific of our own GPU specifications. We merely followed the idea of maximizing the patch size, at the cost of reducing the batch size to unity. Setting the number of epochs primarily followed time considerations (an epoch refers to one cycle through the full training dataset). While increasing the epochs allows the weights to converge longer and therefore could improve the results, the training time can become prohibitively long. The optimal number of epochs is generally considered to be between 100 and 250. While the patch size and the epoch count have significant impacts on the results, it is always possible to interrupt the training process with a set of parameters and resume the training with another set. Therefore, setting a parameter wrongly is not necessarily critical.

Following recommendations [22], the network was trained in 150 epochs, with a batch size of 1 and a patch size of 512. The training phase took ~10 h of computation time on an Nvidia Quadro K2200 (640 CUDA Cores, 4GB GPU memory size, NVIDIA, Santa Clara, CA, USA). After the training phase, the network served to segment all slices in the volume.

Furthermore, the distinct binary U-Net classifiers were trained for the two gradient directions. The same gradient images had been used in the variational segmentation approach. A total of 14 equally spaced slices from the manual segmentation dataset were used to train the neural network. These two networks were trained faster, in about 4 h each (200 epochs, patch size of 64, and batch size of 64). The resulting segmentations were post-processed and merged as before (Section 5).

Eventually, a median filter (size of 3 × 17 × 17 voxels) and removal of small isolated regions (volume <10^5^ voxels) was applied to the segmented volume, as minor post-processing steps. Some details on the median filter adjustment are described in Section 7. The results of the deep learning segmentation approach are presented in Figure 7d–f.

We conclude that the accuracy of the segmentation is mainly dependent on the training dataset. That can be seen in Figure 7, where we compare the result of neural network segmentations, in which the neural networks were trained on either 6 or 14 slices.

All steps of the deep learning segmentation procedures are summarized in Algorithm 4a–b.

**Algorithm 4a: Deep Learning Segmentation Procedure.**

(1) Extract a subset of the manual segmentation (1/25 slices, 14 total), as the labelled set. Corresponding slices are extracted from the raw image, to constitute the learning set. Alternatively, that same learning set could be independently annotated to produce the labelled set instead, for instance in Dragonfly (4.1, Object research systems (ORC) inc, Montreal, Canada).
(2) Choosing options of data augmentation (here, orthogonal rotations and vertical and horizontal mirrors).
(3) Choosing a neural network architecture (U-Net in our case) and setting appropriate training parameters (here, batch size 1, patch size 512, epoch count 150).
(4) Train the neural network.
(5) Use the neural network to segment the full raw image.
(6) Post-processing: Remove small connected components (<= 10^5^ voxels), and median filter with a box structural element of size 3 × 17 × 17.

**Algorithm 4b: Deep Learning Segmentation with Prior Orientation Analysis.**

(1) Do steps 2–3 for warp and fill directions (independently).
(2) Analyze orientation using Algorithm 2.
(3) Segment the image using Algorithm 4a.
(4) Join warp and fill images using function *join_images* with threshold parameters 10^5^ (code in Appendix A).

## 7. Results

Numerical comparison of the manual and automatic segmentation images is presented in Table 1. The Table shows a difference in matrix and tow volume fractions, as well as voxel-wise Dice similarity of the segmentation images (under the segmentation error column). The Dice similarity is the number of different voxels, divided by the total number of voxels (only images with equal size are compared here). The difference between the variational segmentation and the manually labeled volume is 7.33%, whereas for deep learning errors are 4.73% and 7.5% for the segmentation of raw (DL Raw) and gradient images (DL Gradient), respectively.

From the volume fraction values presented in Table 1, we remark that all methods have a close ratio of warp/fill volumes. However, the variational method alone yields a relatively smaller matrix volume fraction. This shrinkage of the matrix region can partly be explained by the downsampling performed. Indeed, the downsampled image could have failed in preserving sharp geometries. On the other hand, it is not prohibitive to use such segmentation for the further processing, because the sharp edge regions can be naturally reconstructed on the meshing stage in case of the correctness of tows geometry. Furthermore, the manual segmentation could have overestimated the matrix volume, because it uses linear interpolation to create tow surfaces from a set of interface contours points (manually picked points over the input µ-CT image).

In Figure 7, the automatic segmentation slices obtained by different methods (Figure 7c–f) are compared to the ground truth (Figure 7b) and to the raw image (Figure 7a). From the images as well as from Table 1, one can conclude that the deep learning approach gives the most accurate results, even though it did not require pre-processing.

**Figure 8:**
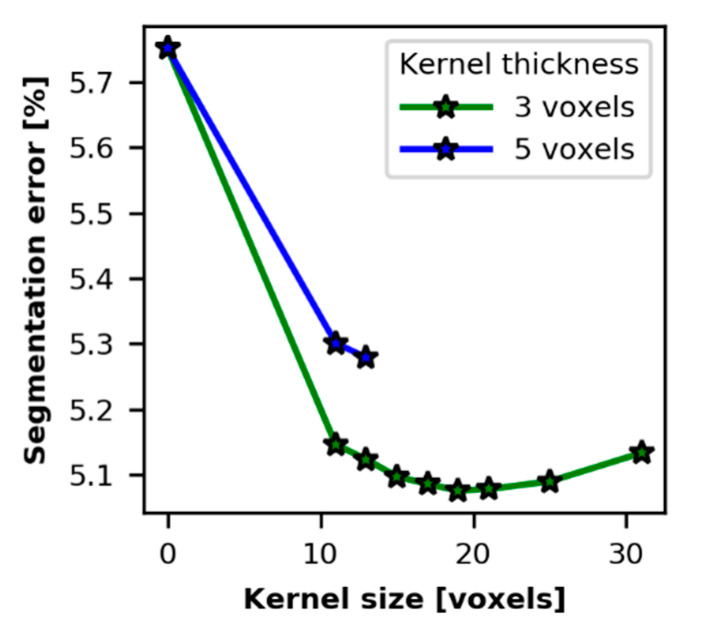
Impact of kernel size of the median filter on the voxel-wise segmentation error.

To increase the efficiency of the segmentation cleaning step, the median filter kernel size was heuristically optimized. Tests were performed on a segmentation of the raw image by an “early” neural network (training set of 14 slices, 200 epochs, and patch size of 64 voxels). Compared to the manual segmentation, this segmentation had an error of 5.75%. The segmentation was cleaned by a median filter with kernel size [ t, w, w ], where size t corresponds to the out-of-plane direction and takes value 3 or 5, and w∈[11, 31] . Selectively, 10 cleaning tests with different kernel sizes were performed. The values of the resulting segmentation error (voxel-wise Dice similarity) for these tests are presented in Figure 8. The minimum error (5.08%) was obtained for the kernel size (3, 19, 19). Moreover, the plots show that the kernel “thickness” increasing from 3 to 5 voxels decreases the segmentation accuracy. The segmentation errors presented in Table 1 were obtained for the kernel size (3, 17, 17). For example, the deep learning segmentation obtained by “final” training on 14 slices with the patch size 512 (Figure 7f) had an initial error of 5.14%. After the cleaning, the error decreased to 4.73%. The used value w=17 is not necessarily optimal for different segmentation tests, but an independent value was chosen for a more adequate comparison of the results. The cleaning is often a compromise between noise removing and features preserving. Based on these tests, we can conclude that, the kernel size [ 3, w, w ], w∈ [11, 31] ensures efficiency of the median filter, applied to the given type of noisy segmentation.

## 8. Discussion

Despite their differences, both approaches have a common important characteristic, which makes them applicable for such kind of challenging images. Both of them use global information to classify a given voxel. The variational approach optimizes the segmentation over the whole 3D image domain. Instead, the U-Net architecture of neural networks specifically follows a contracting path (where the resolution of a patch is gradually decreased), before an expanding path (where the resolution of a downsampled patch is gradually increased back to original), doing so, spatial information and feature information are given varying importance throughout progression within the network. However, we saw that qualitatively, both methods yielded satisfyingly good segmentations. The main limitation of the variational approach is the prior retrieval of the fiber orientation, a complex task by itself.

The fiber orientation analysis can also be used further, for instance, to evaluate the structure tensor approach [12], which is the usual method to obtain texture orientation. This could serve to generalize our orientation analysis to images with more than two preferred tow orientations. Here, however, the difference between the anisotropy levels of matrix and tows is too small for an accurate identification of the matrix regions by the structure tensor approach. Furthermore, we had already retrieved both orientations of interest from the gradient components, and further analysis was not needed. Another possible way of developing the orientation analysis method further (for more complex tow architectures), would be to analyze the morphological gradients calculated along line structure elements that are rotated over a predefined set of inclination angles.

It is worth to mention that the morphological gradient yields a better contrast (Figure 4c) for the vertical orientation of the structure element, than for the horizontal one (Figure 4d). This could be explained by a difference in fiber volume fraction distribution within the warp and fill tows. The difference between warp and fill tows’ total volume, obtained by manual and automatic segmentation (see results of Section 7), also points to that. Another possible influence on the gradient is the material sample position during the X-ray scanning process, which may exhibit an anisotropic point spread function.

The variational segmentation cannot be considered fully automatic, due to the “manual” fitting of the α and λ parameters. Here, the parameters were qualitatively selected. However, more advanced, fully automatic approaches are possible, for instance by exploiting prior knowledge. One possible criterion for automatic parameter fitting would be to enforce that an optimal segmentation (after thresholding and cleaning) should minimize the grayscale standard deviation of the input image, within its sub-regions. Moreover, prior knowledge about the tows’ volume fraction can be used. Once set, these parameters are suitable for the segmentation of other CT images of the material with similar acquisition parameters.

When it comes to the deep learning segmentation and despite plausible results overall, a concern arose about the dimensionality of the algorithm. Indeed, in its current version, Dragonfly software is only able to classify 2D images. Used (in-plane) cross-sections do not account for information present in the other directions. However, Figure 2a shows that at least two different directions can complement each other greatly. Therefore, we speculate that in future works, a segmentation using a 3D capable neural network could further improve the quality of the results. Here the reasonable question appears: “2D slices of which direction of the 3D image should be used for the segmentation?” We tested the approach only for the in-plane direction because they have a stronger anisotropy compared with the side view slices. On the other hand, the manual segmentation of the side view slices (for the training dataset preparation) is easier than in-plane view.

Finally, we could wonder about the impact of the learning set’s cardinality on the deep learning segmentation result. Having a full manually segmented dataset is a luxury that allowed us to use a relatively high number of slices in the learning set. However, one could argue that the effort in manually labeling 14 slices might outweigh the gain in accuracy. We have also tried the segmentation with only six slices in the learning set. Although these results should be mitigated by the relatively low effort that went into labeling the learning set from scratch, the segmentation that resulted from that is relatively accurate (see comparison with the reference dataset in Section 7). Therefore, a decent segmentation result can be achieved also with less amount of manually prepared training data.

Comparing the variational and the deep learning approaches, it is noticeable that for the same input images (from fiber orientation analysis), both methods give us quantitatively similar segmentation (Figure 7c–b): 7.33% error for the variational segmentation and 7.50% for the deep learning. Adding that the variational segmentation result was obtained for downsampled images, one can conclude about the close efficiency of both methods applied to the pre-processed data. However, the application of the deep learning approach to the raw data provides much better accuracy. Furthermore, the deep learning approach produces a neural network, which can be reused to segment new µ-CT data, even in the case of a complex dataset with limited texture.

## 9. Conclusions

µ-CT images of continuous carbon fiber reinforcements composites with plain-woven architecture have been segmented by different novel methods explained in this work. The challenge of the processing is the poor quality of the µ-CT images of the given material, even at the highest possible resolutions, i.e., low signal-to-noise ratio and contrast. Two segmentation approaches have been used: variational method and deep learning. The fiber orientation analysis was performed based on the computation of the morphological gradient. The orientation analysis gives different quality depending on tows direction. This indicates a difference in local volume fraction between warp and fill tows, and/or an impact of the µ-CT scanning setup on the fiber gradients (due to the anisotropy of the material). Compared to manual segmentation of the dataset, the segmentation methods show decent results, even for particularly noisy µ-CT data. The most accurate result was obtained by the deep learning approach, applied to unprocessed µ-CT images and with 14/357 slices in the training data set. However, training U-net segmentation requires manual annotation of the training dataset and more computational power than the other methods. Nevertheless, once trained, the neural networks can be reused for the segmentation of other µ-CT datasets, provided similar acquisition parameters.

The low segmentation errors show that all segmentation approaches are sufficiently accurate to be used for the generation of a realistic textile geometry starting from raw µ-CT data. After the separation of individual tows, the segmentation can also be used for extraction of statistical information about meso-scale geometry for the following generation of an idealized shape model.

## Figures and Tables

**Table 1:** Comparison of the manual and automatic segmentations.

	Volume Fraction (%)			Segmentation Error (%)
	Matrix	Warp Tows	Fill Tows	
Mumford–Shah	9.94	43.54	46.53	7.33
DL Gradient (14 slices)	14.5	41.7	43.8	7.50
DL Raw (6 slices)	15.4	40.1	44.5	6.79
DL Raw (14 slices)	13.9	42.3	43.8	4.73
Manual	15.5	41.2	43.3	-

## Appendix A

Python implementation of removing small isolated islands from a binary image and joining binary images with different labels.

import numpy as np

import scipy.ndimage as ndi

from skimage.morphology import watershed

def remove_small(image, min_size):

  “““The remove all non-zero regions with less than min_size voxels.

  “““

  labels, _ = ndi.label(image)

  count = np.bincount(labels.ravel ())

  is_large = (count >= min_size)

  is_large[0] = False #background

  return (is_large[labels]).astype(image.dtype)

def join_images(images, min_size_zero = 0):

  “““Join binary images and optionally remove small zero regions.

  “““

  images = np.stack(images).astype(np.uint8)

  images[images > 0] = 1

  images_sum = np.sum(images, axis = 0)

  is_fill = images_sum != 1

  is_zero = images_sum = 0

  if min_size_zero > 1:

    is_zero = remove_small(is_zero, min_size_zero)

  for i in range(1, images.shape[0]):

    images_sum + = images[i] * i

  images_sum[is_fill] = 0

  distance = ndi.distance_transform_cdt(is_fill,

    return_distances = False, return_indices=True)

  images_sum[:] = images_sum[distance[0], distance[1], distance[2]]

  images_sum[is_zero] = 0

  return images_sum
