# Correction: Sinchuk et al. Variational and Deep Learning Segmentation of Very-Low-Contrast X-ray Computed Tomography Images of Carbon/Epoxy Woven Composites. *Materials* 2020, *13*, 936

**DOI:** 10.3390/ma15228168

**Published:** 2022-11-17

**Authors:** Yuriy Sinchuk, Pierre Kibleur, Jan Aelterman, Matthieu N. Boone, Wim Van Paepegem

**Affiliations:** 1 Department of Materials Science and Engineering, Faculty of Engineering and Architecture, Ghent University, Technologiepark Zwijnaarde 46, 9052 Zwijnaarde, Belgium; 2 Department of Environment, Faculty of Bioscience Engineering, Ghent University, Coupure Links 653, 9000 Gent, Belgium; 3 Center for X-ray Tomography (UGCT), Ghent University, Proeftuinstraat 86, 9000 Gent, Belgium; 4 Department of Telecommunications and Information Processing–Image Processing and Interpretation, Faculty of Engineering and Architecture, Ghent University—IMEC, Sint-Pietersnieuwstraat 41, 9000 Gent, Belgium; 5 Department of Physics and Astronomy, Faculty of Sciences, Ghent University, Proeftuinstraat 86, 9000 Gent, Belgium

We would like to change the authors’ affiliation on the recent published paper [1] from:

Yuriy Sinchuk ^1,^*, Pierre Kibleur ^2^, Jan Aelterman ^3^, Matthieu N. Boone ^4^ and Wim Van Paepegem ^1^

^1^ Department of Materials Science and Engineering, Faculty of Engineering and Architecture, Ghent University, Technologiepark Zwijnaarde 46, 9052 Zwijnaarde, Belgium; wim.vanpaepegem@ugent.be

^2^ Department of Environment, Faculty of Bioscience Engineering, Ghent University, Coupure Links 653, 9000 Gent, Belgium; pierre.kibleur@ugent.be

^3^ Department of Telecommunications and information processing, Faculty of Engineering and Architecture, Ghent University, Proeftuinstraat 86, 9000 Gent, Belgium; jan.aelterman@ugent.be

^4^ Department of Physics and astronomy, Faculty of Sciences, Ghent University, Proeftuinstraat 86, 9000 Gent, Belgium; matthieu.boone@ugent.be

* Correspondence: yuriy.sinchuk@ugent.be

to the correct version, as follows:

Yuriy Sinchuk ^1,^*, Pierre Kibleur ^2,3^, Jan Aelterman ^3,4,5^, Matthieu N. Boone ^3,5^ and Wim Van Paepegem ^1^

^1^ Department of Materials Science and Engineering, Faculty of Engineering and Architecture, Ghent University, Technologiepark Zwijnaarde 46, 9052 Zwijnaarde, Belgium; wim.vanpaepegem@ugent.be

^2^ Department of Environment, Faculty of Bioscience Engineering, Ghent University, Coupure Links 653, 9000 Gent, Belgium; pierre.kibleur@ugent.be

^3^ Center for X-ray Tomography (UGCT), Ghent University, Proeftuinstraat 86, 9000 Gent, Belgium; jan.aelterman@UGent.be (J.A.); matthieu.boone@UGent.be (M.N.B.)

^4^ Department of Telecommunications and Information Processing–Image Processing and Interpretation, Faculty of Engineering and Architecture, Ghent University—IMEC, Sint-Pietersnieuwstraat 41, 9000 Gent, Belgium

^5^ Department of Physics and Astronomy, Faculty of Sciences, Ghent University, Proeftuinstraat 86, 9000 Gent, Belgium

* Correspondence: Yuriy.Sinchuk@UGent.be

The authors apologize for any inconvenience caused and state that the scientific conclusions are unaffected. This correction was approved by the Academic Editor. The original publication has also been updated.
